# Structure and energy based quantitative missense variant effect analysis provides insights into drug resistance mechanisms of anaplastic lymphoma kinase mutations

**DOI:** 10.1038/s41598-018-28752-9

**Published:** 2018-07-13

**Authors:** Jianzong Li, Yue Huang, Miaomiao Wu, Chuanfang Wu, Xin Li, Jinku Bao

**Affiliations:** 1 Key Laboratory of Bio-Resource and Eco-Environment of Ministry of Education, College of Life Sciences, Sichuan University, Chengdu, 610065 Sichuan P. R. China; 2 College of Computer Science, Sichuan University, Chengdu, 610207 Sichuan P. R. China; 3 State Key Laboratory of Oral Diseases, National Clinical Research Center for Oral Diseases, West China Hospital of Stomatology, Sichuan University, Chengdu, China; 4 State Key Laboratory of Biotherapy/Collaborative Innovation Center for Biotherapy, West China Hospital, Sichuan University, Chengdu, 610041 China

## Abstract

Anaplastic lymphoma kinase (ALK) is considered as a validated molecular target in multiple malignancies, such as non-small cell lung cancer (NSCLC). However, the effectiveness of molecularly targeted therapies using ALK inhibitors is almost universally limited by drug resistance. Drug resistance to molecularly targeted therapies has now become a major obstacle to effective cancer treatment and personalized medicine. It is of particular importance to provide an improved understanding on the mechanisms of resistance of ALK inhibitors, thus rational new therapeutic strategies can be developed to combat resistance. We used state-of-the-art computational approaches to systematically explore the mutational effects of ALK mutations on drug resistance properties. We found the activation of ALK was increased by substitution with destabilizing mutations, creating the capacity to confer drug resistance to inhibitors. In addition, results implied that evolutionary constraints might affect the drug resistance properties. Moreover, an extensive profile of drugs against ALK mutations was constructed to give better understanding of the mechanism of drug resistance based on structural transitions and energetic variation. Our work hopes to provide an up-to-date mechanistic framework for understanding the mechanisms of drug resistance induced by ALK mutations, thus tailor treatment decisions after the emergence of resistance in ALK-dependent diseases.

## Introduction

Anaplastic lymphoma kinase (ALK), a member of the superfamily of insulin receptor protein-tyrosine kinases, was characterized by the identification of a 2;5 chromosomal translocations in anaplastic large-cell lymphoma (ALCL) cell line^1^. This chromosomal rearrangement generates nucleophosmin (NPM)-ALK fusion protein that has a constitutively activated ALK kinase domain^2^. In addition to NPM-ALK fusion protein, the echinoderm microtubule-associated protein-like 4 (EML4)–ALK fusion detected in NSCLC is the most widely identified^3^. It has been indicated that the ALK fusion proteins play an important role in driving tumorigenesis^2,3^. In contrast to fusion proteins, activation of the full-length ALK is normally regulated by extracellular ligand-binding domain. The full-length ALK consists of an extracellular ligand-binding domain (residues 19–1038), a transmembrane domain (residues1039–1059) and an intracellular tyrosine kinase domain (residues 1116–1392). Experimental genetic evidences indicate that mutated full-length ALK plays an important role in multiple carcinomas, such as neuroblastoma and thyroid cancer, but the mechanisms have not been illuminated very clearly^4–7^.

ALK has been validated as a therapeutic molecular target for the treatment of ALK-rearranged cancer. Substantial efforts among academia and pharmaceutical industry have been made to develop effective ALK inhibitors. Nowadays, crizotinib, ceritinib and alectinib have been approved by the US Food and Drug Administration (US. FDA) for the treatment of patients with advanced “ALK-positive” NSCLC^8–13^. Considerable small-molecular inhibitors targeting ALK are currently in clinical trials, such as AP26113^14^ and lorlatinib (PF-06463922)^15^. However, the rapid emergence of inevitable drug resistance is occurring worldwide, endangering the efficacy of chemotherapy involving these drugs. Generally, different ALK inhibitors actually result in the emergence of resistance to ALK inhibitors that is characterized by different mechanisms. Crizotinib is the first-generation ALK inhibitor, resistance to this drug occurs in patients who initially benefited from target therapies. It is reported that about one third of resistance cases are related to the diverse mutations in EML4-ALK fusion protein^16^. Acquired secondary ALK resistance mutations to the crizotinib include I115ITins, L1152P/R, C1156Y/T, I1171T/N/S, F1174C/L/V, V1180L, L1196M, G1202R, S1206C/Y, E1210K, or G1269A/S^17^. Ceritinib and alectinib are the second-generation ALK inhibitors that are developed to overcome the resistance to the first generation ALK inhibitors, but resistant mutation to these drugs are also inevitably reported. Resistant mutations to ceritinib include I115ITins L1152P/R, C1156Y/T, I1171T/N/S, F1174C/L/V, and G1202R. Resistant mutations to alectinib include I1171T/N/S and G1202R. Among which L1196M gatekeeper mutation is the most common resistance mutation to crizotinib^17–19^. The hotspot mutations F1174 (mutated to L, S, I, C or V) in ALK kinase domain are identified in about 85% of the cases with ALK mutations. G1202R is located at the solvent front of the ALK kinase domain and exhibits broad-spectrum resistance to all ALK inhibitors. There may be some other potential resistance harboring in primary ALK mutations. Although the functional research for these mutations are very limited, more and more experimental evidences show that they play an important role in tumorigenesis and may possess potential effects on ALK targeting therapy^20–22^.

Numerous studies have been performed to dissect the mechanisms of drug resistance to ALK inhibitors^7,23–25^. It has been widely acknowledged that the drug-resistant mutations cause drug resistance by re-inducing kinase activation and signaling despite the presence of the inhibitors. These mutations can hinder the inhibitor binding to ALK, alter the kinase’s conformation, and/or alter the ATP-binding affinity of the kinase^7,23,25^. It has been suggested that evolving paradigms exist in cancer drug resistance and contribute to the evolution process of tumor clones in response to the selection pressure by drug treatments^26,27^. Some interesting works have evaluated the influence of subtle mutations on the shifts of the energetics and function of proteins as well as specific stability-function tradeoffs in the evolution processes of enzymes^20^. Comprehensive quantitative profiling of mutational effects on drug resistance is increasingly important in tailoring treatment decisions after the emergence of resistance in ALK-depend diseases given the promise of novel molecular targeted therapies. Of particular interest is whether the mechanisms that mediate drug resistance in protein are caused by a few mutations occurring in distal regions. Here, we focus on exploring diverse mechanisms of drug resistance induced by secondary or primary mutations in ALK and hope to find some common shared features. In addition, a comprehensive profile of drugs against ALK mutations has been constructed to analyze the energetic variation mediated by mutations, thus providing insights into the molecular basis of drug resistance from a structural perspective. We show how an integration of structural transition and energy variation moves us towards a better understanding of why biological molecules have the properties that they do, such as the emergence of acquired drug resistance.

## Results and Discussions

### Stability landscape of all possible mutations in ALK kinase domain

**Figure 1:**
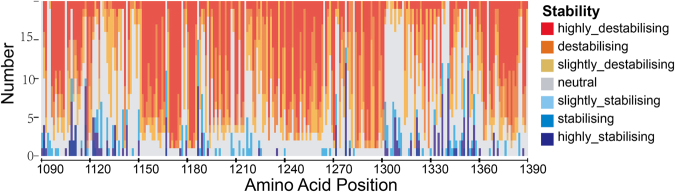
Effect of mutations on ALK kinase domain stability. All 19 possible mutations along with the synonymous mutations that the residue mutates to itself at each position in ALK kinase domain are colored on a vertical bar in terms of their stability relative to wide-type ALK. The values of ΔΔ*G*_*fold*_ are binned into seven categories: highly stabilizing (ΔΔ*G*_*fold*_ < −1.84 kcal·*mol*^−1^) and highly destabilizing (ΔΔ*G*_*fold*_ > 1.84 kcal·*mol*^−1^); stabilizing (−1.84 kcal·*mol*^−1^ < ΔΔ*G*_*fold*_ < −0.92 kcal·*mol*^−1^) and destabilizing (0.92 kcal·*mol*^−1^ < ΔΔ*G*_*fold*_ < 1.84 kcal·*mol*^−1^); slightly stabilizing (−0.92 kcal·*mol*^−1^ < ΔΔ*G*_*fold*d_ < −0.46 kcal·*mol*^−1^) and slightly destabilizing (0.92 kcal·*mol*^−1^ < ΔΔ*G*_*fold*_ < 1.84 kcal·*mol*^−1^); and neutral (−0.46 kcal kcal·*mol*^−1^ < ΔΔ*G*_*fold*_ < 0.46 kcal·*mol*^−1^).

In order to estimate the stability effect of all possible mutations of each residue of the ALK kinase domain, the stability changes (ΔΔ*G*_*fold*_) were computed using FoldX. As shown in Fig. 1, most of the mutations (2730 of 4940 = 55.26%) have moderate thermodynamic stability effects, the values of ΔΔ*G*_*fold*_ of these mutations range from −1.84 kcal·*mol*^−1^ to 1.84 kcal·*mol*^−1^. 2170 mutations (43.93%) showed highly destabilizing (ΔΔ*G*_*fold*_ > 1.84 kcal·*mol*^−1^). A handful of mutations are found to have strongly stabilizing effect on the structure of ALK kinase domain, only 40 mutations (0.81%) show that the values of ΔΔ*G*_*fold*_ are less than −1.84 kcal·*mol*^−1^, most of these mutations are located in or close to the kinase activation segment of ALK, the detailed data was provided in Supplementary Table S1. Experimental evidences have demonstrated that residues in the active sites of the enzyme are characterized by intrinsic destabilizing due to the high energy state, and the replacement of these residues can actually increase the stability of the enzyme^28–30^.

**Figure 2:**
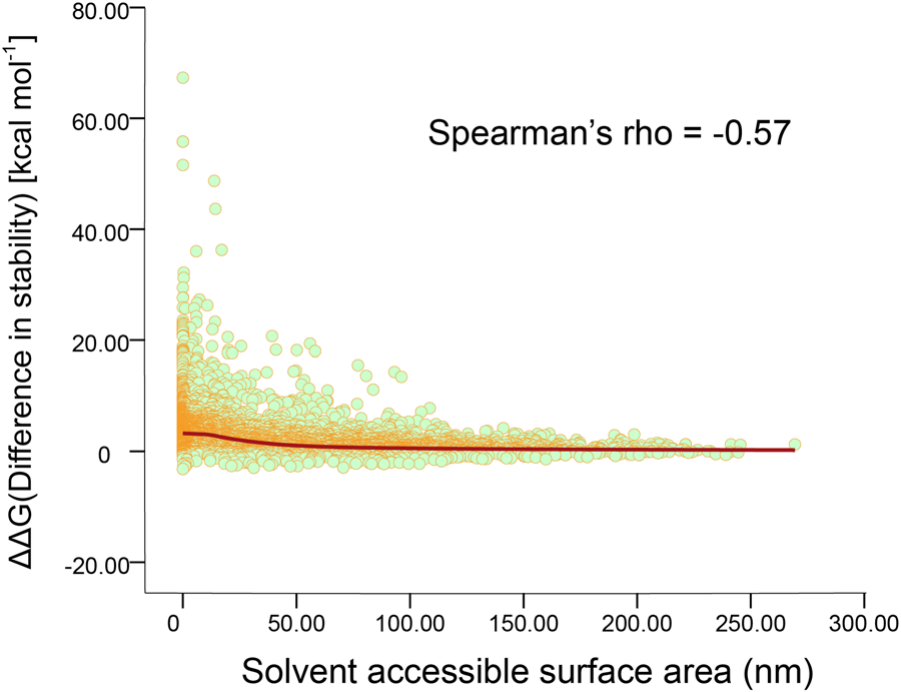
Scatter plot of the correlation between solvent accessible surface area (SASA) and difference in stability (ΔΔ*G*_*fold*_) of ALK mutations.

Interest in quantitative analysis of the correlation between the degree of surface exposure and stability effect of residues led us to further investigate the relevance of the stability effect of mutation and the solution accessible surface area (SASA) of each residue. Buried residues are not accessible to the solvent inside a cell, therefore their SASA usually equal zero or closes to zero. Here, SASA of each residue was calculated to estimate the surface exposure level using FreeSASA^31^. Then we simply used Spearman’s rank correlation coefficient (Spearman’s rho) to investigate how well the relationship between ΔΔ*G*_*fold*_ and SASA could be described using a monotonic function. As shown in Fig. 2, the Spearman’s rho equaled −0.57, indicating that moderate negative correlation existed between ΔΔ*G*_*fold*_ and SASA. Highly destabilizing mutations (ΔΔ*G*_*fold*_ > 1.8 kcal·*mol*^−1^) were more likely to occur in residues that possessed smaller SASA. If mutations occur in buried residues, their ΔΔ*G*_*fold*_ can be very high. Mutations with larger SASA show smaller volatility of stability effects, and these mutations are usually neutral or slightly destabilizing.

It has been well known that surface residues are not important for protein stability since their interactions with the solvent should be similar in the unfolded and native states^32^. Here, results showed that the changes in stability mediated by the mutation were related to the solvent-accessible area of residues. For example, surface residue Met1290 possessed highest SASA (208.26 nm), and mutations occurring in this position had moderate effects on protein stability, most of their ΔΔ*G*_*fold*_were lower than 0.92 kcal·*mol*^−1^, only M1290V was highly destabilizing (3.79 kcal·*mol*^−1^). The SASA of surface residue Lys1114 was 172.55 nm, the changes in stability mediated by the mutation in this position were also insignificant, the average of ΔΔ*G*_*fold*_ value in this position was 0.17 kcal·*mol*^−1^, no mutation occurring in the site was stabilizing or destabilizing. Once residues possess SASA over 70 nm, highly destabilizing or stabilizing mutations are unlikely to occur in these sites (Supplementary Table S2). Results reveal that mutations occur in exterior regions have moderate effects on protein stability, while mutations that occurr in interior regions with low-level surface exposure are more likely to be destabilizing.

### Effects of observed ALK mutations on protein evolution and drug resistance

Theoretically, there may be incalculable possible mutational forms in processes of evolution, but ALK has to satisfy diverse constraints, including reasonable folding, thermodynamic stability and solubility, to maintain its specific function^29,33,34^. Only few ALK mutations are compatible with the constraints. Here, 145 observed mutations occurring in ALK kinase domain were collected from Uniprot^35^, COSMIC^36^ and ClinVar^37^ database to investigate the stability-function tradeoffs followed by these mutations and their evolutionary features underlying drug treatment condition. Most of observed mutations are associated with diseases and play an important role in the pathogenesis of neuroblastoma, anaplastic thyroidcarcinoma and NSCLC^20–22,38,39^, 131 mutations are the original primary mutation in neuroblastoma and lung adenocarcinoma cell lines, and 14 mutations are acquired as secondary mutations occurring independently or dependently in individual NSCLC cell line (Supplementary Table S3).

**Figure 3:**
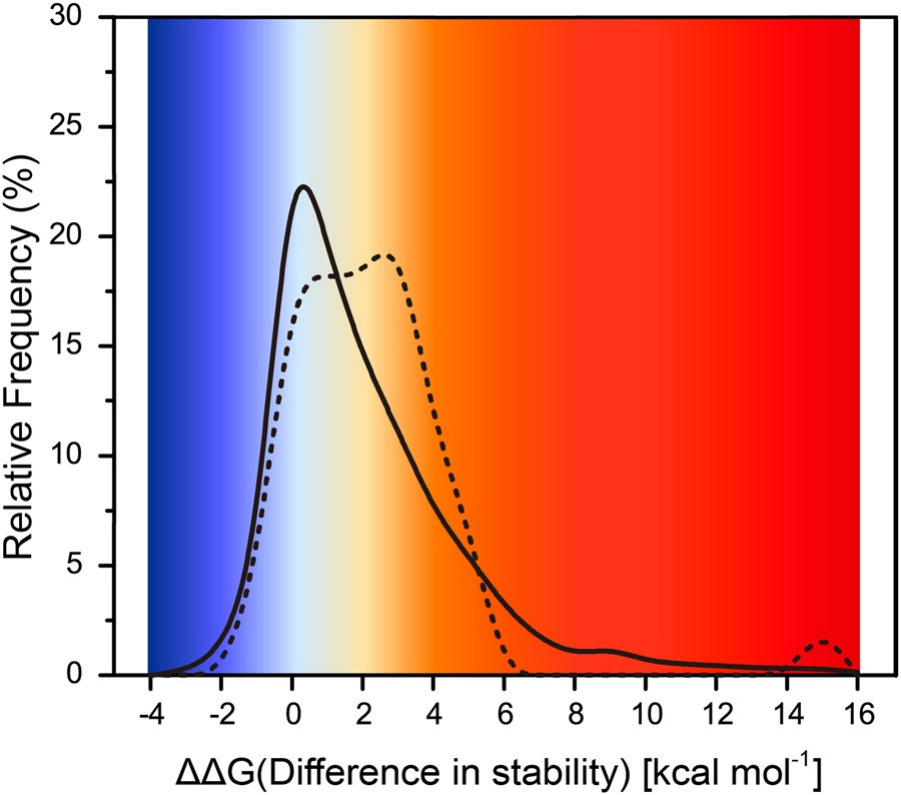
Distribution of stability effects of all possible simulated mutations and those observed in clinic. The distribution of stability changes arising from mutations observed in tumor cell (dashed line) stands in contrast to that of all possible simulated mutations (solid line). The probability distributions shown here are obtained by kernel smoothing of the original data

The distribution of the ΔΔ*G*_*fold*_ values of all possible mutations of ALK kinase domain and observed mutations occurring during the evolution, or rather, tumorigenesis was computed. As can be seen in Fig. 3, the distribution of stability effects of all these possible mutations is unimodal and has its largest peak close to a ΔΔ*G*_*fold*_ of zero, suggesting that the simulated mutations appear to be neutral or have moderate effects on the protein thermodynamic stability. In contrast to all possible mutations, the distribution of the ΔΔ*G*_*fold*_ values of observed mutations follows a multimodal distribution and has an excess of slight destabilizing than possible mutations. As can be seen, it has two slight distinct peaks including a peak of high destabilizing whose values centered at 3.12 kcal·*mol*^−1^ and a smaller peak close to zero.

Although highly destabilizing mutations are unlikely to occur in nature conditions, mutations that observed in clinic appear to be highly destabilizing. The stability-function tradeoffs hypothesis suggests that substitution of key residues in enzymes decreases the stability, resulting in the increasing activity of enzyme^40^. Studies of enzyme steady-state kinetic parameters of ALK showed that F1174L mutant attained 86% of the activity of the phosphorylated ALK, and R1275Q mutant attained 28% of the activity of the phosphorylated ALK^41^. The ΔΔ*G*_*fold*_ values of F1174L mutant was 1.40 kcal·*mol*^−1^ and the ΔΔ*G*_*fold*_ values of R1275Q mutant was 1.90 kcal·*mol*^−1^. In addition, mutations occurring in Fhe1174 loci (mutated to Ile, Leu, Ser or Val) also attained higher ΔΔ*G*_*fold*_ values, they were able to relax the steric clash with F1245 occurring in the catalytic loop and thus facilitated activation^22,23^. These mutations have been described in several diseases as mentioned above, as well as drug resistance in NSCLC. Our results are consistent with the “stability-function” hypothesis^42^. Mutations occurring within or near key regulatory regions can dramatically increase the activity of the enzyme owing to the intervention of destabilizing mutations.

Furthermore, the ΔΔ*G*_*fold*_ values of total 21 reported drug resistance mutations were investigated here. In particular, we found that ALK drug resistance mutations presented significantly more destabilizing, most of which attained higher ΔΔ*G*_*fold*_ values than 1.84 kcal·*mol*^−1^. They were the main forces to form the peak of high destabilizing whose values centered at 3.12 kcal·*mol*^−1^ in the distribution of stability effects. Their ΔΔ*G*_*fold*_ values ranged from −1.02 kcal·*mol*^−1^ (E1210K/S1206C) to 11.06 kcal·*mol*^−1^ (L1152P). Mean of the ΔΔ*G*_*fold*_ values was 3.03 kcal·*mol*^−1^ and the median was 3.07 kcal·*mol*^−1^, see Table 1. Rare drug resistance mutations are stabilizing, only four mutations can slightly stabilize the structure, including the gatekeeper L1196M mutation (−0.16 kcal·*mol*^−1^), L1198F mutation (−1.19 kcal·*mol*^−1^) and two double mutations E1210K/S1206C and L1198F/C1156Y (−0.26 kcal·*mol*^−1^). The gatekeeper mutation L1196M is the most common crizotinib-resistance mutant identified in NSCLC. The replacement of leucine by methionine, which has comparable stabilizing effects on the ALK structure as well as identical electronic properties and steric demands. Our previous study showed that the loss of a non-polar binding energy interaction between the leucine side chain and the methyl substituent of crizotinib might account for the resistance against crizitinib when leucine was mutated to a methionine^43^. The mechanisms of which other drug resistance mutations facilitate activation of ALK in the context of drug treatment conditions are currently unclear. ALK inhibitors approved by the US. FDA are all type I1/2 inhibitors that bind to an inactive protein kinases with the DFG-in conformation^44,45^, including cizotinib, ceritinib and alectinib. It has been demonstrated that mutations could facilitate the transition between inactive and active conformations and increased the stability of the active conformation. Here, our analysis suggests that the activation of ALK is increased by the substitution with destabilizing mutations, creating the capacity to confer drug resistance inhibitors.

**Table 1:** Effects of reported resistance mutations to the ALK inhibitors approved by the US.

Mutation		ΔΔG_fold_ kcal·*mol*^−1^	±*SD*	ΔE	ALK inhibitors^a^				
					Crizotinib	Ceritinib	Alectinib	Brigatinib	Lorlatinib
Primary mutation	L1152P	11.06	0.024	−5.84	**+**	**+**			
	I1171T	3.07	0.026	−2.72	**+**		**+**		
	I1171N	3.18	0.030	−4.94	**+**		**+**		
	I1171S	2.83	0.009	−4.54	**+**		**+**		
	F1174C	4.22	0.027	−6.24	**+**	**+**			
	F1174S	5.14	0.069	−7.26	**+**	**+**			
	V1180L	0.96	0.027	−3.98	**+**		**+**		
	L1198F	−1.19	0.024	−4.14					**+**
	S1206C	3.23	0.350	−5.28	**+**				
Secondary mutation	L1152R	3.17	0.409	−4.86	**+**	**+**			
	C1156Y	4.47	0.634	−4.41	**+**				
	F1174L	1.36	0.185	−2.54	**+**	**+**			
	L1196M	−0.16	0.039	−4.82	**+**				
	L1198P	1.46	0.058	−7.18	**+**				
	G1202R	2.88	0.140	−5.76	**+**	**+**	**+**	**+**	
	D1203N	3.85	0.149	−4.46	**+**				
	S1206Y	2.72	0.236	−5.64	**+**				
	G1269A	0.52	0.376	−1.41	**+**				
	G1269S	2.92	0.556	−1.72	**+**				
	E1210K/S1206C	−1.02	0.273	−8.25				**+**	
	E1210K/D1203N	0.90	0.215	−7.78				**+**	
	L1198F/C1156Y	−0.26	0.449	−9.17					**+**

FDA or in clinical testing.

^a^Plus sign indicates that the mutation is reported resistance mutation to corresponding ALK inhibitor.

Interestingly, S1206C mutant has been reported as crizotinib-resistant mutation, but the double ALK mutant (S1206C/E1210K) does not show resistance to crizotinib anymore. The resistance to ALK inhibitors shifts from crizotinib to brigatinib, a new ALK inhibitor that is currently under clinical testing. C1156Y mutant has been reported as crizotinib-resistant mutation and ceritinib-resistant mutation, but the double mutant (L1198F/C1156Y) confers resistance to lorlatinib and causes sensitization to crizotinib^19^. C1156Y, D1203N and S1206C are highly destabilizing mutations underlying our results, L1198F and E1210K are stabilizing mutations that can increase the thermodynamic stability of ALK, their ΔΔ*G*_*fold*_ values are −1.19 kcal·*mol*^−1^ and −0.46 kcal·*mol*^−1^, respectively. It has been demonstrated that the maintenance of the protein’s stability is a strong constraint to evolution^46^, drug resistance mutations are actually subject to the constraints^47^. In the scenario of double mutations S1206C/E1210K, C1156Y/L1198F and D1203N/E1210K, the stability effects of high destabilizing mutations on ALK structure are constantly pruned by the stabilizing mutations.

**Figure 4:**
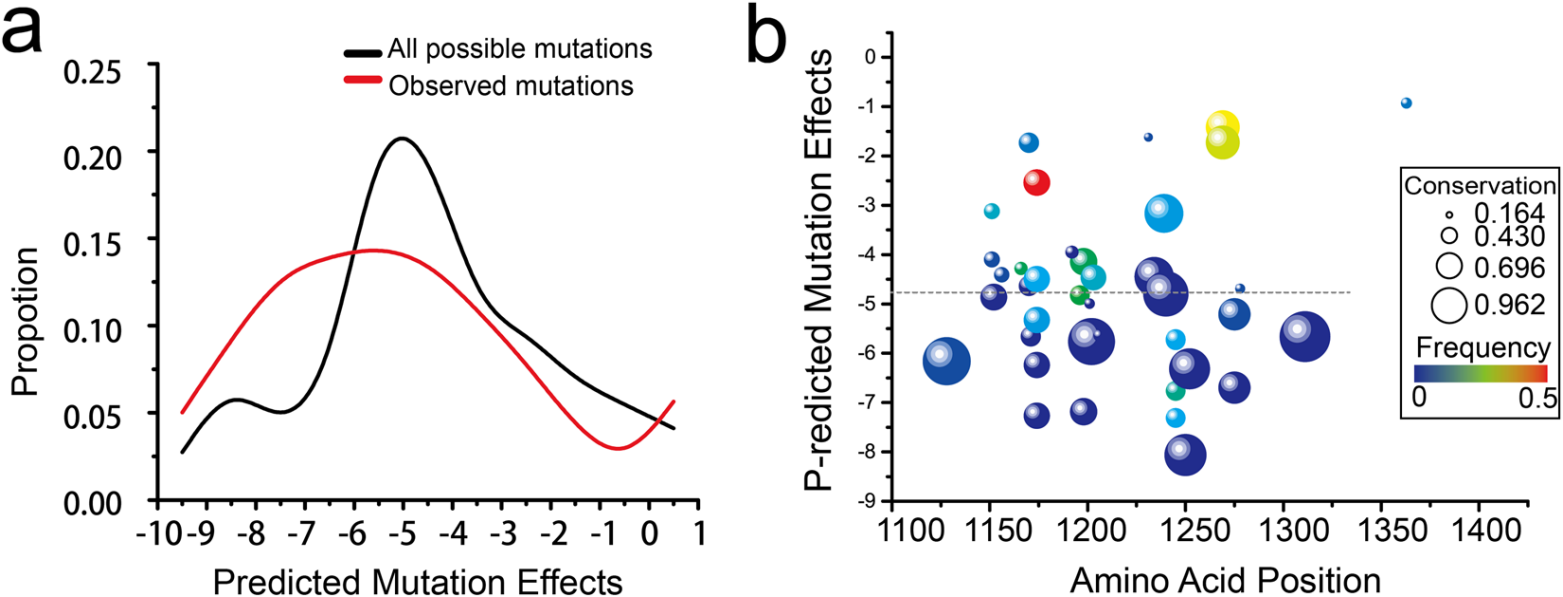
The distribution of mutational effects (Δ*E*) of ALK mutations. (**a**) The distribution of mutation effect scores of all possible mutations and observed mutation calculated by Evmutation software. (**c**) Scatter plot of mutation effects of pathogenic mutations. The dash line indicates the median value of these scores (Δ*E* = −4.86). The mutation effects shown here are obtained by bubble and color mapped of the original data.

Accounting for the evolutionary forces that may cause drug resistance can be important for providing reasonable treatment strategies, we employed computational tools to estimate whether resistance mutations in the kinase domain were likely to be benign or damaging using comparative evolutionary considerations. The estimations of the mutation effects (ΔE) of ALK mutations were presented in Fig. 4, the detailed data were provided in Supplementary Table S4. The distributions of mutation effect scores of all possible and observed mutations showed similar variation tendency, the largest peak of ΔE values of the observed mutations were slightly lower than that of all possible mutations. Most observed mutations are identified as pathogenic or likely pathogenic mutations. Only 5 are mutations are not specified, their effects on phenotypes are unclear. The median value of the pathogenic mutations’ effect scores is −4.86 taking into account of epistasis, indicating that most of the observed mutations in ALK kinase domain are possibly damaging. The ΔE values of drug resistance mutations ranged from −7.26 (F1174S) to −1.41 (G1269A), the median value was −4.84 and the mean value of the ΔE was −4.64, see Table 1. Results reveals that disease-associated drug resistance mutations are not likely to be tolerated in the kinase domain of ALK. However, current paradigm of drug resistance mutations indicates that mutations occur prior to treatment are likely to be benign or natural, thus they can escape from the fixing of evolutionary constraints^48^. We further examined whether secondary drug resistance mutations were more likely to be damaging than primary mutations in the ALK kinase domain. We found their ΔE values were very comparable, no remarkable distinction was observed. In the scenario of double mutations, their ΔE values were all higher than that of the constituent single mutations, see Table 1. Although double mutations attain moderate effects on the thermodynamic stability of ALK kinase domain, their effects on the protein fitness are likely to be possibly damaging. Taken together, our results imply that the evolution constraint limits the adaptation of ALK to drug treatment contexts through additional amino acid changes that can shift the drug resistance properties without dramatic effects on the overall thermodynamic stability, resulting in more deleterious effects and eventually failure of novel drug treatments.

### Comprehensive profiles for ALK inhibitors against drug resistance mutations

**Figure 5:**
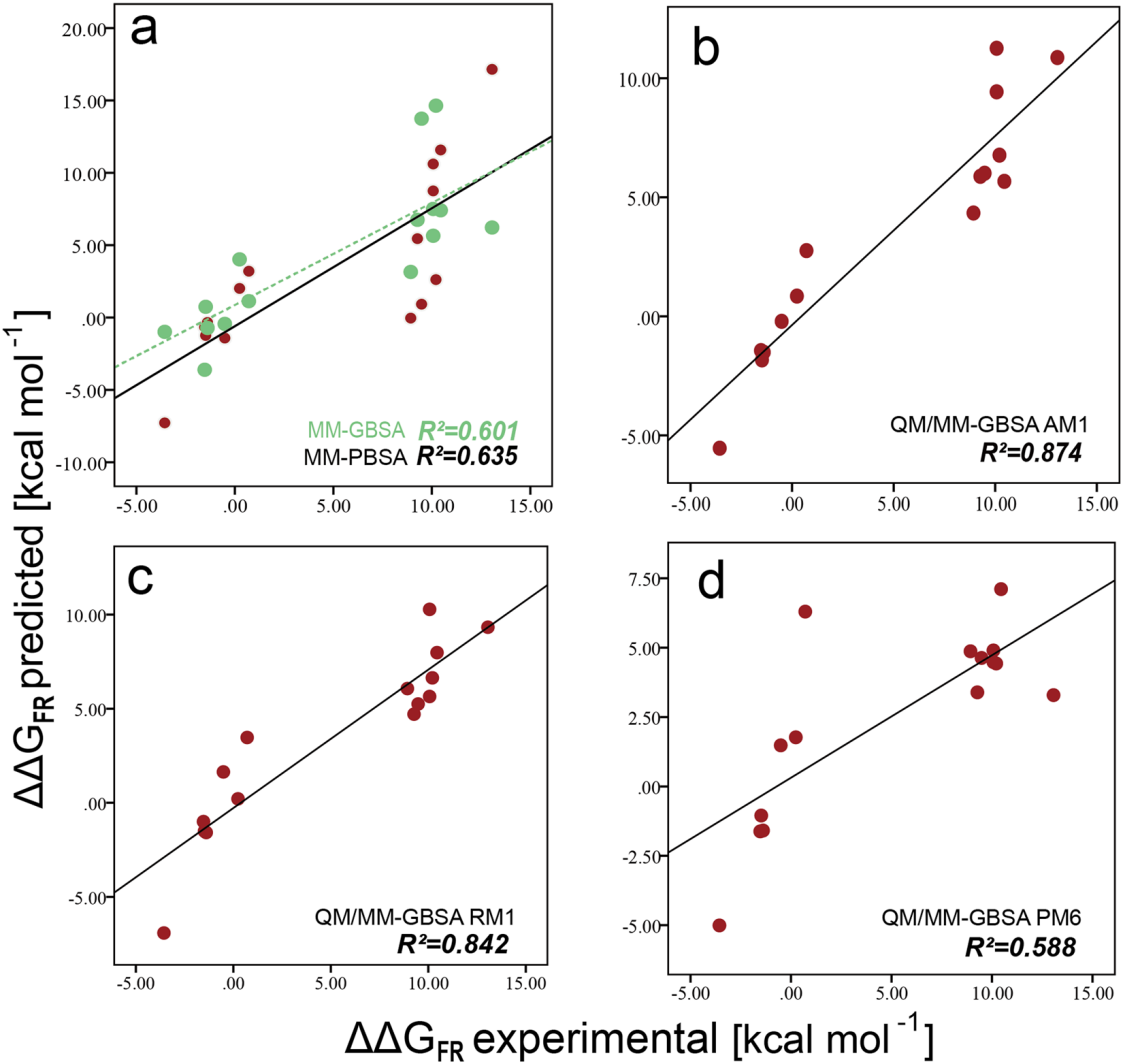
Inhibition constants of ALK Inhibitors with Wild-type and mutant ALK kinase. Predicted FR energies (ΔΔ*G*_*FR*_ predicted) vs experimental FR energies (ΔΔ*G*_*FR*_ experimental) for inhibitors with ALK and mutations. (**a**) MM-GBSA (0.601); (**b**) MM-PBSA (0.635); (**c**) AM1 (0.874); (**d**) RM1 (0.842); (**d**) PM6 (0.588).

To characterize the effect of drug resistance mutations on ALK inhibitor binding, we built a systematic extensive energy profile of drugs against ALK drug resistance mutations by means of MM/QM-GBSA calculation. In order to test the reliability of different schemes in calculating inhibitor binding affinity change of ALK mutations, 15 pairs of the experimental mutation energies (${\rm{\Delta }}{\rm{\Delta }}{G}_{FR}\,experimental$) and predicted mutation energies (${\rm{\Delta }}{\rm{\Delta }}{G}_{FR}\,predicted$) were utilized. ${\rm{\Delta }}{\rm{\Delta }}{G}_{FR}\,experimental$ can be estimated by the formula: ${\rm{\Delta }}{\rm{\Delta }}{G}_{FR}\,experimental$ = RT·ln·(${K}_{i}\,mutant/{K}_{i}\,wildtype),\,\,$where R = 1.9872 × 10^−3^ kcal·*K*^−1^·*mol*^−1^, and T = 300 K^49,50^. The experimental data of ${K}_{i}mutant$ and ${K}_{i}wildtype$ were collected from studies contributed by Alice T. Shaw and co-workers^19^. Five different schemes were used to calculate the values of ${\rm{\Delta }}{\rm{\Delta }}{G}_{FR}\,predicted$ (Supplementary Table S5). ${\rm{\Delta }}{\rm{\Delta }}{G}_{FR}\,predicted$ can be derived utilizing the binding free energy of the inhibitor against the ALK mutations (Δ*G*_*mt*_) minus that of wild type (Δ*G*_*wt*_). The correlation between ${\rm{\Delta }}{\rm{\Delta }}{G}_{FR}\,experimental$ and ${\rm{\Delta }}{\rm{\Delta }}{G}_{FR}\,Predicted$ can be reflected by coefficient of determination (*R*^2^). The closer *R*^2^ approaches 1, the better positive correlation that the two variables possess, thus indicating the better results that the calculating scheme has. As shown in Fig. 5a, the green points were the results calculated by the MM-GBSA method, while the red points stood for the results calculated by MM-PBSA, which both used the molecular mechanics (MM) theory. The *R*^2^ of MM-GBSA was 0.601, while that of MM-PBSA was 0.635, indicating that the latter scheme had the better prediction for the values of ${\rm{\Delta }}{\rm{\Delta }}{G}_{FR}\,predicted$ as they were more matched with ${\rm{\Delta }}{\rm{\Delta }}{G}_{FR}\,experimental$. The semi-experiential Hamiltonian methods based on QM/MM-GBSA theory were used here, including AM1, RM1 and PM6^51–53^. As shown in Fig. 5, the *R*^2^ of these schemes were 0.874 (AM1), 0.842 (RM1) and 0.588 (PM6), respectively, indicating that the AM1 scheme possessed the best performance to predict the inhibitor binding affinity change among the five schemes. Thus, by utilizing the AM1 method for calculation, considerable reliability was assured for the further analysis.

**Figure 6:**
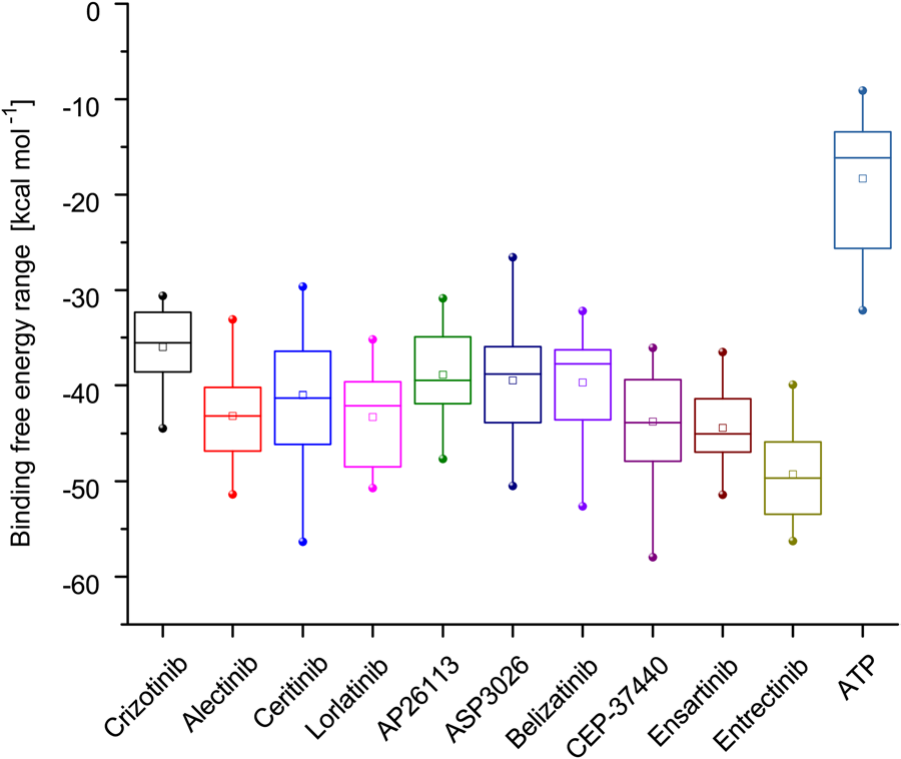
Range of binding free energies of 10 ALK inhibitors and a substrate ATP against 21 reported drug resistance mutations occurring in ALK kinase domain.

Range of binding free energies (Δ*G*_*mt*_) of the 10 ALK inhibitors along with ATP against 21 reported drug resistance mutations were shown in the box-plot (Fig. 6). Obviously, the averages and medians of the Δ*G*_*mt*_ values of the 10 inhibitors were way lower than those of ATP (average as −18.07 kcal·*mol*^−1^, median as 15.92 kcal·*mol*^−1^). Among the inhibitors, the first-generation ALK inhibitor crizotinib had the overall minimal binding affinity (with the average Δ*G*_*mt*_ as −35.74 kcal·*mol*^−1^ and the median Δ*G*_*mt*_ as −35.05 kcal·*mol*^−1^) against ALK as it was the first small molecule inhibitor approved by US. FDA for the treatment of ALK positive NSCLC and considerable acquired resistances acted upon it. The second-generation inhibitors include alectinib and ceritinib, which both manifested better binding affinity than crizotinib. Lorlatinib, AP26113, ASP3026, belizatinib, CEP-37440, ensartinib and entrectinib all belong to the third-generation inhibitors, of which entrectinib had the overall maximal binding affinity (with the average Δ*G*_*mt*_ as −49.07 kcal·*mol*^−1^ and the median Δ*G*_*mt*_ as −49.58 kcal·*mol*^−1^) among all 10 inhibitors, suggesting it may be a promising inhibitor against the drug resistance mutations of ALK kinase domain. Generally, the second-generation and the third-generation inhibitors had exhibited better binding affinity than the first-generation inhibitor.

**Figure 7:**
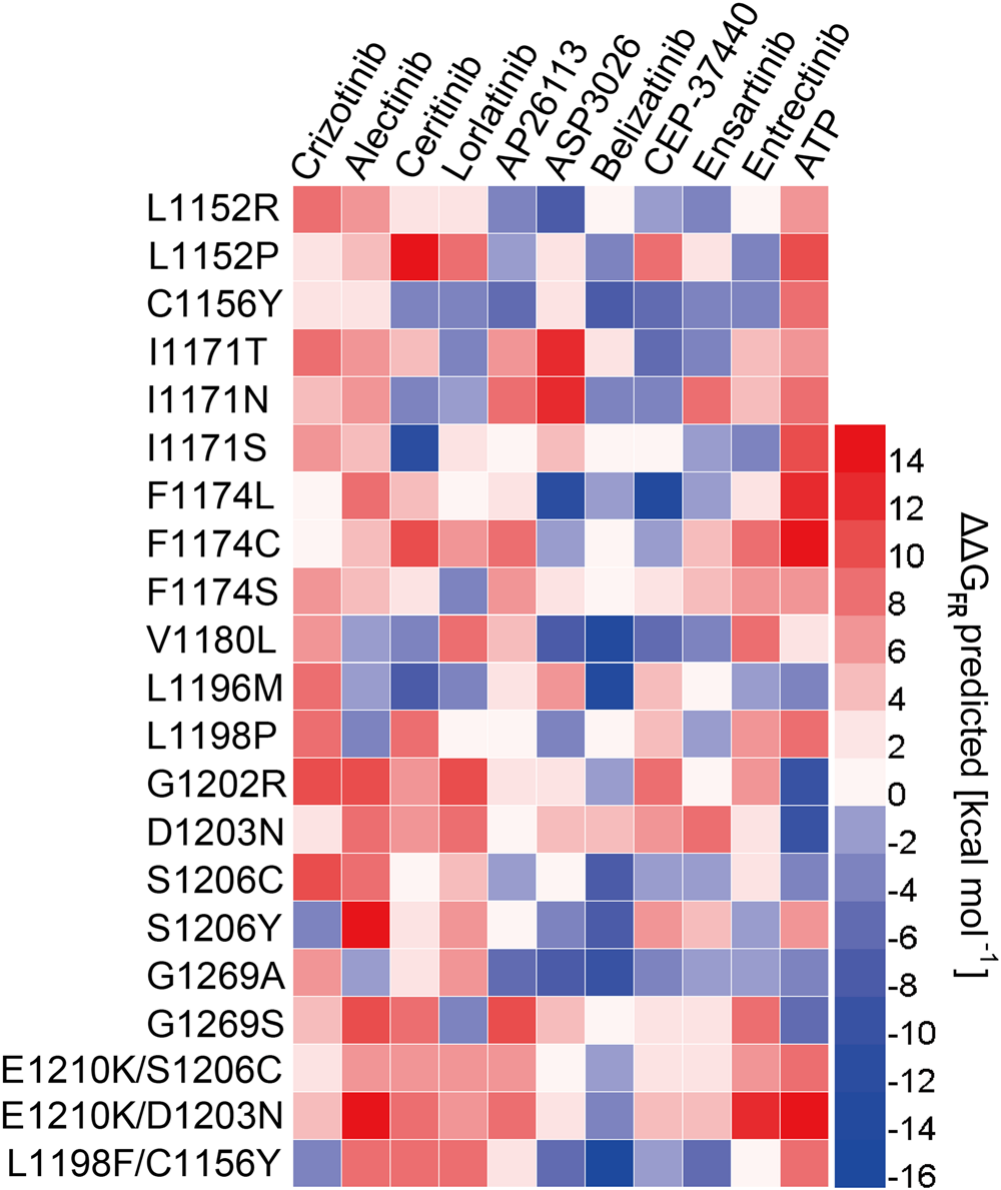
Heat map of the mutation energy profile of 10 ALK inhibitors as well as a substrate ATP against 21 reported drug resistance mutations occurring in ALK kinase domain.

The ${\rm{\Delta }}{\rm{\Delta }}{G}_{FR}\,predicted$ of the 10 inhibitors as well as ATP against 21 drug resistance mutations in ALK kinase domain were calculated by the QM/MM-GBSA AM1 scheme (Supplementary Table S6). The results were shown as the heat map in Fig. 7. As can be seen in Fig. 7, the majority of the ${\rm{\Delta }}{\rm{\Delta }}{G}_{FR}\,predicted$ values stayed within the range from −6 kcal·*mol*^−1^ to 6 kcal·*mol*^−1^ while a few ${\rm{\Delta }}{\rm{\Delta }}{G}_{FR}\,predicted$ had considerably great absolute values, indicating the violent affinity changes of these mutations compared with the wild type. The minimum of ${\rm{\Delta }}{\rm{\Delta }}{G}_{FR}\,predicted$ occurred at the mutation energy of the inhibitor belizatinib against the double-point mutation L1198F and C1156Y, suggesting that the mutation gained much better affinity towards the inhibitor compared to the wild type. The maximum of ${\rm{\Delta }}{\rm{\Delta }}{G}_{FR}\,predicted$ occurred at the mutation energy of the inhibitor ceritinib against the single-point mutation L1152P, indicating that ceritinib bound to the mutation much less closely than to the wild-type ALK and strong drug resistance may occur.

**Figure 8:**
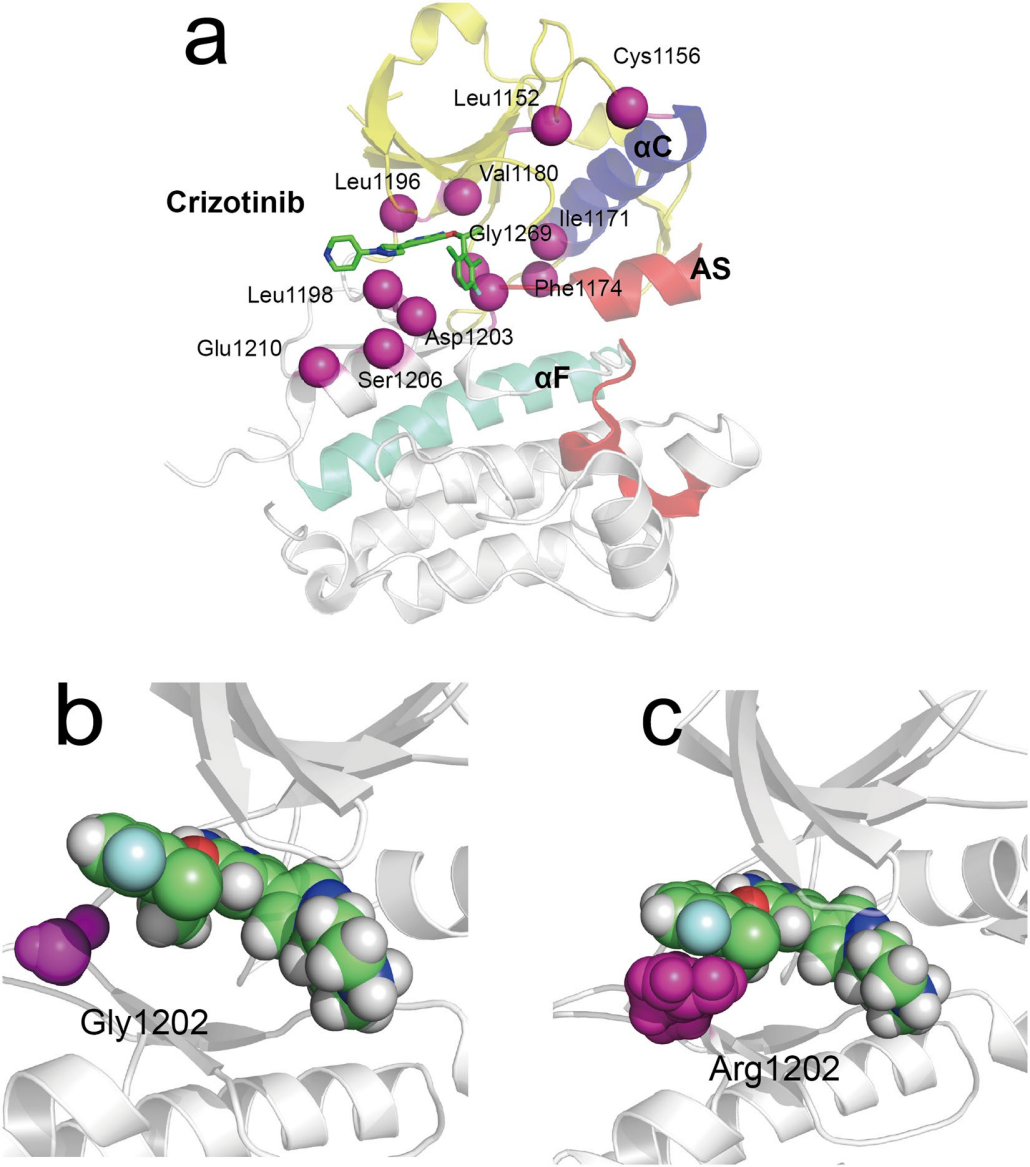
Structural Basis for drug resistance to crizotinib. (**a**) The location of ALK drug resistance mutations. AS refers to activation segment. The co-crystal structures of crizotinib bound to the wild type (panel b) and Arg1202 mutant (panel c).

As for the first-generation inhibitor crizotinib, the first drug resistance reported was the L1196M gatekeeper mutation^54^. It alters the residue at the bottom of the ATP-binding pocket and impairs inhibitor binding^25^. ΔΔ*G*_*FR*_ predicted of crizotinib against L1196M was 8.59 kcal·*mol*^−1^, suggesting that crizotinib had lost considerable affinity towards the ALK kinase domain after ALK mutated. The loss of a non-polar binding energy interaction (Δ*G*_*surf*_) might account for the resistance against crizitinib when leucine was mutated to a methionine^43^. Here, binding free energy of crizotinib against L1196M calculated by QM/MM-GBSA demonstrated that electrostatic interaction (Δ*E*_*ele*_ was more reduced than Δ*G*_*surf*_ (Supplementary Table S7). G1202R is highly resistant to first- and second-generation inhibitors^25^ and it also occurs in the ATP-binding pocket, see Fig. 8. In the mutation, glycine was replaced with arginine, causing the cleft becoming narrower and hindering the binding of crizotinib with increased steric clash. The ΔΔ*G*_*FR*_ predicted values of crizotinib against G1202R was 10.08 kcal·*mol*^−1^, suggesting that considerable binding affinity was lost. The loss of van der Waals interaction energy (Δ*E*_*vdw*_), about 8.78 kcal·*mol*^−1^, was the dominant factor in causing such variation.

Other reported single-point drug resistance mutations against crizotinib include L1152P/R, C1156Y, I1171T/N/S, F1174C/L/V, V1180L, S1206C/Y and G1269A/S^25^, where V1180L and G1269S located in the ATP-binding pocket. L1152P, L1152R and C1156Y affect residues adjacent to the N-terminus while F1174C/L/V affect residues adjacent to the C-terminus of the αC helix^16,18,54,55^. The ΔΔ*G*_*FR*_ predicted values of 13 mutations were positive, indicating worse affinity after mutations. While only that of S1206Y was negative, which was −2.97 kcal·*mol*^−1^. S1206Y belongs to the solvent-front mutations which impair drug binding likely through steric hindrance^16,17^. The Δ*E*_*ele*_, ΔE_*vdw*_, Δ*G*_*surf*_ and quantum mechanics energy (Δ*G*_*qm*_) provided favorable contribution to the inhibitor bindings. The unfavorable polar solvation energy (Δ*G*_*gb*_) partially impaired these favorable factors. Especially, we found these drug mutations also attained weaker Δ*E*_*ele*_ than that of wild type, except S1206C, G1269S and C1156Y/L1198F. The Δ*E*_*ele*_ is significant in ligand-receptor binding and plays an key role in the stability of biomolecules. The destabilizing mutations can obviously reduce electrostatic interactions which turn in changing the ligand binding affinity. It should be noticed that the double-point mutation L1198F and C1156Y had a negative ΔΔ*G*_*FR*_ predicted value (−3.51 kcal·*mol*^−1^) while the ΔΔ*G*_*FR*_ predicted values of single-point mutation C1156Y was 2.36 kcal·*mol*^−1^. Previous study suggest that L1198F enhanced binding to crizotinib, negating the effect of C1156Y and re-sensitizing resistant mutations to crizotinib^19^. The Δ*E*_*ele*_ of C1156Y/L1198F (−146.84 kcal·*mol*^−1^) was slightly lower than that of wild type (−144.18 kcal·*mol*^−1^), its Δ*E*_*vdw*_ (−46.57 kcal·*mol*^−1^) was also slightly lower than wild type (−45.45 kcal·*mol*^−1^). Their Δ*G*_*qm*_were very comparable. The Δ*E*_*qm*_ of wild type was −21.21 kcal·*mol*^−1^ and C1156Y/L1198F was −21.68 kcal·*mol*^−1^.

The mutation L1196M is the most common mutation that confers crizotinib resistance^16^, which promoted the development of the second-generation inhibitors (such as alectinib and ceritinib)^56^. The mutation energies of alectinib and ceritinib against L1196M were −0.04 kcal·*mol*^−1^ and −6.54 kcal·*mol*^−1^ respectively while that of crizotinib was 9.3 kcal·*mol*^−1^, suggesting the better affinity of alectinib and ceritinib. Besides L1196M, ceritinib was also reported to overcome inhibitor-bearing ALK mutations (such as I1171T and S1206Y), while some mutations were reported resistant to ceritinib (such as F1174C and G1202R)^57–59^. The mutation energies of ceritinib against these mutations were 5.07 kcal·*mol*^−1^ (I1171T), 3.77 kcal·*mol*^−1^(S1206Y), 10.65 kcal·*mol*^−1^ (F1174C) and 6.51 kcal·*mol*^−1^ (G1202R) respectively. It is not hard to explain why ceritinib is resistant to F1174C, as the ${\rm{\Delta }}{\rm{\Delta }}{G}_{FR}\,predicted$ value was rather high, suggesting that considerable affinity had been lost. However, the ${\rm{\Delta }}{\rm{\Delta }}{G}_{FR}\,predicted$ values of I1171T and S1206Y were all positive while they were sensitive to ceritinib. It seemed strange on the first sight, but if the mutation energies of ceritinib’s competitor (which was ATP) were considered at the same time, then it may not be as paradoxical as it seemed to be. The ${\rm{\Delta }}{\rm{\Delta }}{G}_{FR}\,predicted$ of ATP against I1171T and S1206Y were 7.31 and 6.88 kcal·*mol*^−1^ respectively, which were all higher than those of ceritinib (which were 5.07 and 3.77 kcal·*mol*^−1^ respectively). The affinity changes of ceritinib were not very significant, and that of ATP was more violent. As result, although ceritinib lost some of its affinity towards the mutations, it still showed more affinity than its competitor ATP, thus performing its function as an effective inhibitor towards the mutations. The third-generation inhibitor lorlatinib was developed as a more potent ALK antagonist, which was reported sensitive to L1196M, L1152R, C1156Y, F1174L and S1206Y^60^. Their ${\rm{\Delta }}{\rm{\Delta }}{G}_{FR}\,predicted$ values were not very high and all of them were lower than that of ATP, suggesting its effectiveness as an inhibitor. It should be noticed that although C1156Y is sensitive to lorlatinib, adding L1198F disrupts the binding of drug and leads to resistance^25^. The ${\rm{\Delta }}{\rm{\Delta }}{G}_{FR}\,predicted$ value of lorlatinib against the double mutation L1198F/C1156Y was 8.64 kcal·*mol*^−1^, suggesting that considerable binding affinity was lost and strong drug resistance towards lorlatinib was gained.

From the discussion above, an assumption could be concluded: if the ${\rm{\Delta }}{\rm{\Delta }}{G}_{FR}\,predicted$ value of the inhibitor against the mutation is significantly high (suggesting that considerable affinity was lost), chances are that the mutation may be strongly resistant to the inhibitor. If the ${\rm{\Delta }}{\rm{\Delta }}{G}_{FR}\,predicted$ value is not very high, then it should be compared with that of ATP to determine which (the inhibitor or ATP) has the better affinity towards the mutation. If the inhibitor gains better affinity (has higher ${\rm{\Delta }}{\rm{\Delta }}{G}_{FR}\,predicted$ value) than ATP after mutation, then chances are that the mutation could be sensitive to the inhibitor. Therefore, bold speculations could be made that belizatinib, CEP-37440 and ensartinib could be highly promising inhibitors as they gained overall better affinity towards many drug-resistant mutations compared to ATP. Besides, the mutations D1203N and G1202R could be quite intractable antagonists as many inhibitors fail to gain better affinity than ATP towards them.

### Structure basis for drug resistance mediated by ALK mutations

**Figure 9:**
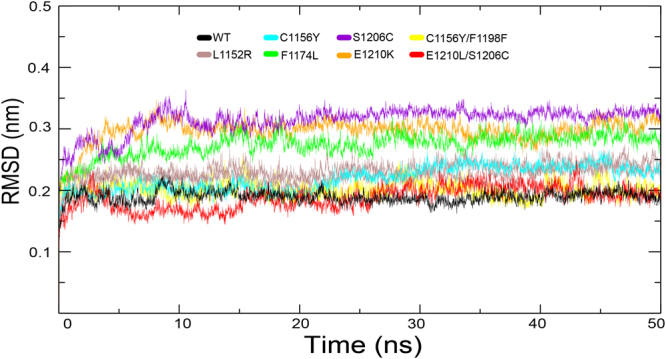
RMSD with respect to the initial structure as a function of time for the various simulations over 50 ns.

Our previous analysis demonstrated that mutations at the active site could inference the steric hindrance and change ligand binding affinity, thus resulting in the emergence of resistance to ALK inhibitors. In addition, increased ATP binding affinity has been discussed for several ALK mutants. However, the structural mechanism by which non-active site mutations confers resistance against ALK inhibitors still remains unknown. Several studies have been conducted to explore the structural mechanism of drug resistance mediated by the mutations occurring in non-active site. They found that these mutations could regulate the dislocation of ALK inhibitors and the indirect conformational changes in the binding cavity^61,62^. To provide a structural framework for understanding drug resistance mutations, 50-ns MD simulations were performed for wild type and mutant systems. The inherent flexibility profile of a protein is a key factor for its full functional activity and ligand binding. The root-mean-square deviation (RMSD) trace was used here to evaluate the dynamics of mutation structure during inhibitor binding, see Fig. 9. It can be seen that the RMSD showed obvious differences between the various simulations. Wild type ALK attained an average of RMSD at 0.197 nm (1.97 Å). The initial 10 ns were excluded as burn-in when average RMSD were calculated. The single mutations showed larger RMSD than wild type system. These were the L1152R, C1156Y, F1174L, S1206Y and E1210K. Their average RMSD values were 2.38 Å, 2.32 Å, 2.86 Å, 3.27 Å and 3.12 Å, respectively. While double mutations C1156Y/L1198F and E1210K/SC1206C attained similar RMSD with wild type. Their average RMSD values were 2.01 Å and 1.98 Å, respectively. The RMSD traces of the double mutations were similar with wild type ALK, indicating that these mutations possessed identical flexibility with wild-type ALK. In previous analysis, we raised that the changed of protein thermostability induced by high destabilizing mutations were fixed by additional stabilizing mutations. Here, results suggested that the flexibility of protein was also constrained by co-evolution of residues.

**Figure 10:**
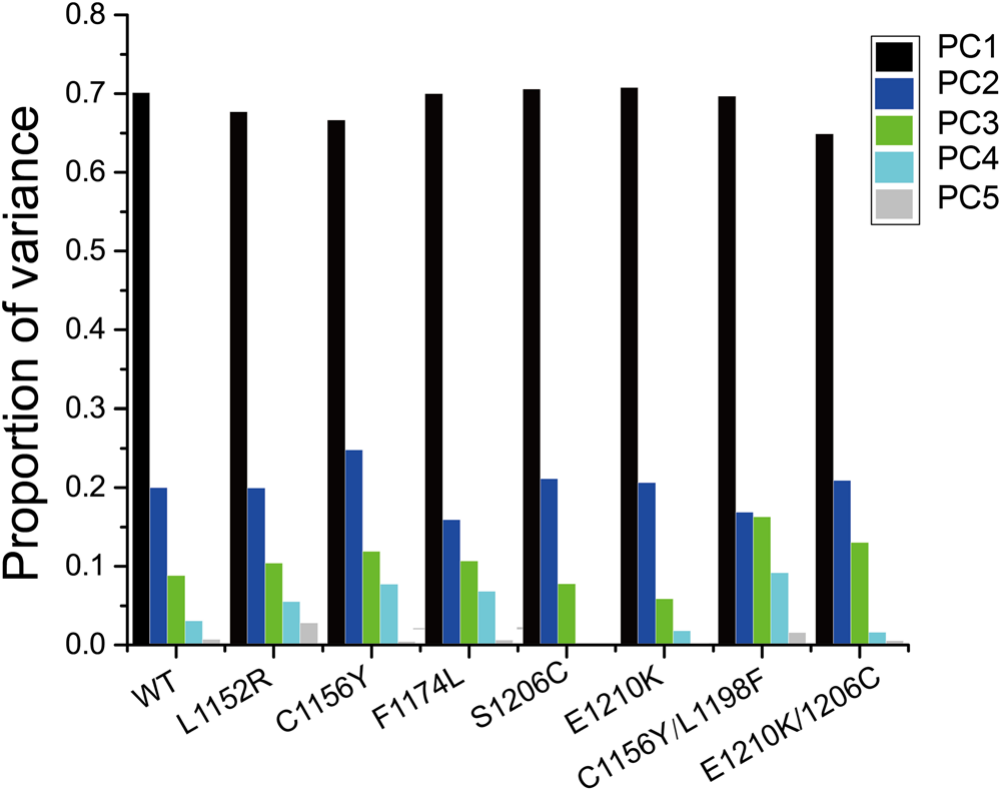
The histogram indicates the variance proportion of each principal component from PC1 to PC5.

**Figure 11:**
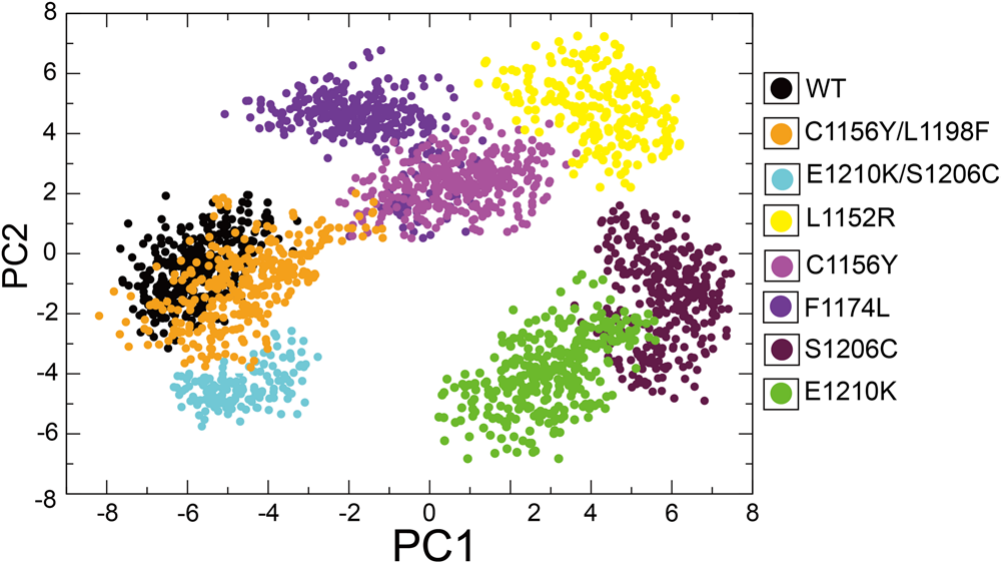
PCA scatter plot along first two principal components, eigenvector 1 (PC1) and eigenvector 2 (PC2) showing difference between the wild type and mutant systems.

It has been well established that the conformational motions activated the dynamical transition of conformational sub-states, resulting in protein relaxation and movements of the ligand within the protein^63,64^. We determined whether the transition could be responsible for the resistance against ALK inhibitors when mutations occur in non-active sites, such as L1152R, C1156Y, F1174L, S1206Y and E1210K. Principal component analysis (PCA) was performed to represent the motion of the native and mutant forms in phase space during crizotinib binding. We extracted eigenvector indexes from the covariance matrix and estimated the correlations between protein motion and eigenvector indexes. The top two dominant principal components PC1 and PC2 accounted for a significant amount of the overall motion, and they can explain more than 85% of the variance together in each case (Fig. 10). The overall motions represented by PC1 and PC2 for wild type and mutant systems were presented in Fig. 11. As shown here, the overall motions between wild-type and most of drug-resistant mutations were extremely diverse. C1156Y showed slightly similar motion with F1174L and S1206C showed slightly similar motion with E1210K. C1156Y/L1198F showed comparable overall motions compared with wild-type as well as E1210K/S1206C, indicating double mutations possessed identical flexibility and similar conformation transition compared with wild-type ALK. This may be one of the reason why double mutations confer sensitization to crizotinib^19^.

**Figure 12:**
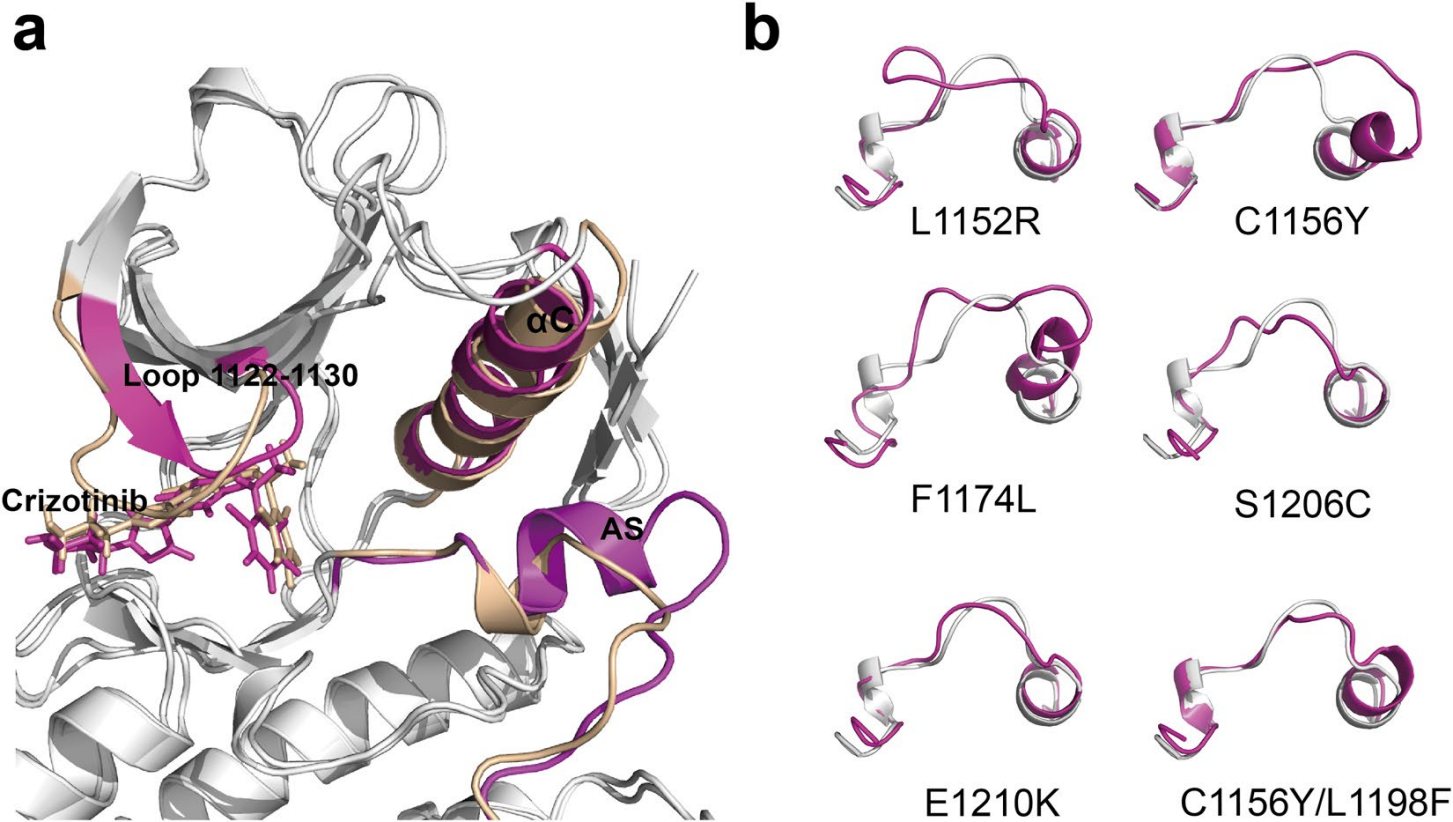
Alignment of structures of wild-type ALK and the non-active sites mutations. (**a**) Comparison of structures of wild-type ALK and C1156Y mutation. C1156Y results in marked conformational changes in activation segment (AS), αC helix and loop 1122–1130. (**b**) Comparison of activation segment structures of wild-type ALK (white) and different mutations (light magenta).

In addition, we compared the average structure of wild-type and mutations during the last 10 ns of the simulation. Figure 12a showed that crizotinib attained an RMSD value of 1.38 Å when the structure of C1156Y mutation aligned to wild-type, confirming that the C1156Y mutation caused conformational changes and ligand motion. Activation segment of ALK is a key regulation component in determining the more active less active kinase state^65,66^. We compared the average structures of activation segment of different mutations with wild-type extracted from the last 10-ns trajectory, see Fig. 12b. The structures of activation segments of the mutations were obviously heterogeneous compared with wild-type. Their activation segment showed more open and incompact conformations than that of wild-type, such as L1152R, C1156Y and F1174L, which were considered as the features of more active state form that allow protein/peptide binding. These mutations are too far to directly block inhibitor binding, their ΔΔ*G*_*fold*_ values were all higher than −1.84 kcal·*mol*^−1^, see Table 1. It is interesting to note that an energy dissipation-cum-signaling mechanism by which distal residues were thermodynamically coupled to activate sites had been established^67–70^. The extent of conformational changes induced by these mutations can be up to over 20 Å^69^, thus resulting in identifiable functional consequences for ligand binding and/or activity of kinase. The propagation of distal mutational perturbation may play a vital role in drug resistance induced by these mutations. Our previous analysis found that highly destabilizing mutations increase kinase activity. Taken together, we suggest that highly destabilizing mutations alter the kinase’s conformation via propagative effect, possibly re-inducing kinase activity despite the presence of inhibitor, in turn causing drug resistance to ALK inhibitors.

## Conclusion

Our primary goal was to quantitatively analyze mutational effects of the ALK drug resistance mutations and provide an up-to-date mechanistic framework for understanding the mechanisms of resistance mediated by these mutations. Firstly, the stability changes (ΔΔ*G*_*fold*_) were computed to estimate the mutational effects of the ligand-free structure of the ALK mutations. The protein fitness of the drug resistance mutations was investigated using comparative evolutionary considerations. We found that drug resistance mutations were significantly more destabilizing, creating the capacity to confer drug resistance to ALK inhibitors. The stability effects of high destabilizing mutations on ALK structure were constantly pruned by the stabilizing mutations. We suggest that co-evolution may limit the ALK’s fitness to drug treatment conditions through additional amino acid changes that can shift the drug resistance properties without dramatic effects on the overall thermodynamic stability, causing failure of drug treatments. Secondly, QM/MM-GBSA calculation was performed to investigate the mutational effects of the drug resistance mutations upon ligand binding, we showed evidence that mutations at the active site inferenced the steric hindrance and changed ligand binding affinity, resulting in the emergence of resistance to the ALK inhibitors. In addition, increased ATP binding affinity had been discussed for several drug-resistant ALK mutations. Thirdly, we explored the conformation transition mediated by drug-resistant mutations. We found that highly destabilizing mutations did alter the kinase’s conformation via propagative effect despite the presence of ALK inhibitors, resulting in the dislocation of the inhibitors and possibly re-inducing kinase activity. Taken together, our work provides comprehensively quantitative analysis for unraveling drug resistance mechanisms of ALK mutations from multiple perspectives.

## Methods

### Structure Preparation

All solved ALK mutation structures were obtained from PDB database. The non-solved *in silico* mutant structures of ALK were generated from a minimized structure of ALK kinase domain (PDB code: 2XP2) using the PyMOL mutagenesis tool. To refine the solved models and the *in silico* mutant, we performed energy minimizations with the GROMACS package using the GROMOS96 force field^71^.

### Stability effect calculation

To compute the free-energy change of the mutations of ALK, FoldX4^72^ was used as a practical tool. Calculations are based on a minimized structure of ALK kinase domain (PDB code: 2XP2). By applying FoldX’s *BuildModel* function, 19 possible point mutations can be introduced to each residue one at a time and 3 independent runs can be carried out calculating respective value of ΔΔG (ΔΔG = ΔG_mut_ − ΔG_wt_), which were then averaged getting the final value ΔΔ*G*_*fold*_. The reported accuracy of FoldX is 0.46 kcal·*mol*^−1^, and ΔΔ*G*_*fold*_ values were then binned into seven categories accordingly, see Fig. 1.

### Mutation effect score calculation

The pairwise model of sequences was computed from a family sequence alignment of ALK using *plmc*. The alignment was in aligned A2M format. The strength of *l*_2_-regularization *λ*_*j*_ on the couplings based on the length of the model was set to 52.2. Other parameters were set as default. Then mutation effect scores (ΔE) were predicted by Evmutation^73^ using epistatic model.

### Molecular Dynamics simulation

In order to investigate the stability of the ALK-ligand complexes, MD simulations were carried out by GROMACS 5.1^71^. By using the pdb2gmx module, the structure files were converted into the topology file with AMBERff99SB^74^ force field and TIP3P water model^75^ applied. As for the ligands’ topology files, they were generated by the antechamber module in AmberTools^76^ with general amber force field^77^ applied. Then, the dodecahedron periodic box was created with the minimal distance between the molecules and the box set to 1.0 nm. The pre-equilibrium steps include system energy minimization, location-constrained pre-simulation, NVT (constant number of particles, volume, and temperature) pre-equilibrium and NPT (constant number of particles, pressure, and temperature) pre-equilibrium, whose purpose is to let the system approach equilibrium as near as possible for the better MD simulations. To be more specific, in the energy minimization period, water molecules along with 0.15 M Na^+^ and Cl^−^ were added into the already energy-minimized system in vacuo to get the new energy-minimized state in solution. In the location-constrained period, the position of the atoms of the receptor-ligand complex remained unchanged while solvent molecules and icons move randomly for further reaching equilibrium. The NVT period lasts for 1000 ps with the system temperature set constantly to 300 K and the sampling time step set to 2 fs. In this period, cutoff method was used to calculate short-range electrostatic interactions and Particle Mesh Ewald method (PME)^78^ was used to calculate long-range electrostatic interactions (rlist = 1 nm, VdW distance = 0.9 nm, cutoff distance = 0.9 nm, and PME order = 4)^79^. Linear Constraint algorithm^80^ was adopted to fix the length of all bonds in the system. In the NPT pre-equilibrium, some parameters (duration, system temperature and time step) stayed the same as those in NVT period, while the system pressure maintains constantly to 1 atm in NPT. Finally, the MD simulation continued for 50 ns to analyze the bonding affinity of the complex system while trajectory information was collected every 10 fs for further analysis.

### Binding affinity calculation

The QM/MM-GBSA approach in Amber was utilized to calculate the binding free energies between the receptors (wild type or mutant) and the inhibitor. Relevant formulas are as follows:

$$\begin{array}{rcl}{\rm{\Delta }}{{G}}_{{mt}} & = & {{G}}_{{mt}}^{{complex}}-({{G}}_{{mt}}^{{receptor}}+{{G}}^{{inhibitor}})\\ {\rm{\Delta }}{{G}}_{{wt}} & = & {{G}}_{{wt}}^{{complex}}-({{G}}_{{wt}}^{{receptor}}+{{G}}^{{inhibitor}})\\ {G} & = & {H}-{TS}\\ {H} & = & {{E}}_{{vdw}}+{{E}}_{{ele}}+{{E}}_{{int}}+{{G}}_{{gb}}+{{G}}_{{surf}}\end{array}$$

Δ*G*_*mt*_ is the interaction free energy of the mutant-inhibitor system, where ${G}_{mt}^{complex}$, ${G}_{mt}^{receptor}$, ${G}^{inhibitor}$ are the free energies of the mutant-inhibitor complex, mutant ALK and the inhibitor. Similarly, Δ*G*_*wt*_ is the interaction free energy of the wildtype ALK-inhibitor system. G stands for the Gibbs free energy, and H, T, S stand for enthalpy (SI unit: joule), temperature (SI unit: kelvin), entropy (SI unit: joule per kelvin) respectively. The terms *E*_*vdw*_, *E*_*ele*_, *E*_*int*_, *G*_*gb*_, *G*_*surf*_ stand for van der Waals interaction, electrostatic interaction, internal energy, polar and non-polar solvation free energy respectively. *G*_*gb*_ is calculated by the modified GB model developed by Onufriev *et al*.^81^. *G*_*surf*_ is estimated by the empirical formula *G*_*surf*_ = γ × *SASA* + β where *γ* and β were set to 0.0072 kcal·*mol*^−1^·${{\rm{\AA }}}^{-2}$ and 0.0 kcal·*mol*^−1^, respectively^82^. From Δ*G*_*mt*_ and Δ*G*_*wt*_, inhibitor affinity change of the ALK (ΔΔ*G*) can be derived as ΔΔG = Δ*G*_*mt*_ − Δ*G*_*wt*_. It is reported that QM/MM-GBSA approach often overestimated the interaction free energies^83^. However, since the change of the interaction free energies (ΔΔ*G*)is what we care and the deviation in ΔΔG is already considerably eliminated by Δ*G*_*mt*_ minus Δ*G*_*wt*_, Thus, the system variation in QM/MM-GBSA approach would not bring about serious impacts to our results. In the QM/MM method, the system was divided into two layers: the inner layer and the outer layer. The inner layer contains the residues involving the hydrogen binding interactions and the residues under mutations, which was treated with the quantum mechanics (QM) theory by applying the semi-experiential Hamiltonian methods. The outer layer consists of the rest of the system, which was treated with the molecular mechanics (MM) theory. As for the entropy, it was calculated by applying a normal-mode analysis at the MM level^84^.

### Principal component analysis

PCA is a useful tool to determine the correlated motions of the residues to a set of linearly uncorrelated variables called principal components. This method is based on the construction of the covariance matrix of the coordinate fluctuations of the simulated proteins. The eigenvectors and eigenvalues are obtained by diagonalizing the covariance matrix, which provides information about correlated motions throughout the protein. *gmx-covar* from GROMACS was used to perform PCA on 50-ns MD trajectories. Overall rotational and translational motions were removed by fitting the protein structure of each time frame to the initial frame.

## Electronic supplementary material

Supplementary Table S1

Supplementary Table S2

Supplementary Table S3

Supplementary Table S4

Supplementary Table S6

Supplementary Table S5

Supplementary Table S7
